# Viral zoonotic risk is homogenous among taxonomic orders of mammalian and avian reservoir hosts

**DOI:** 10.1073/pnas.1919176117

**Published:** 2020-04-13

**Authors:** Nardus Mollentze, Daniel G. Streicker

**Affiliations:** ^a^Medical Research Council–University of Glasgow Centre for Virus Research, Glasgow G61 1QH, United Kingdom;; ^b^Institute of Biodiversity, Animal Health and Comparative Medicine, College of Medical Veterinary and Life Sciences, University of Glasgow, Glasgow G12 8QQ, United Kingdom

**Keywords:** infectious disease, reservoir, surveillance, generalized additive model

## Abstract

The notion that certain animal groups disproportionately maintain and transmit viruses to humans due to broad-scale differences in ecology, life history, and physiology currently influences global health surveillance and research in disease ecology, virology, and immunology. To directly test whether such “special reservoirs” of zoonoses exist, we used literature searches to construct the largest existing dataset of virus–reservoir relationships, consisting of the avian and mammalian reservoir hosts of 415 RNA and DNA viruses along with their histories of human infection. Reservoir host effects on the propensity of viruses to have been reported as infecting humans were rare and when present were restricted to one or two viral families. The data instead support a largely host-neutral explanation for the distribution of human-infecting viruses across the animal orders studied. After controlling for higher baseline viral richness in mammals versus birds, the observed number of zoonoses per animal order increased as a function of their species richness. Animal orders of established importance as zoonotic reservoirs including bats and rodents were unexceptional, maintaining numbers of zoonoses that closely matched expectations for mammalian groups of their size. Our findings show that variation in the frequency of zoonoses among animal orders can be explained without invoking special ecological or immunological relationships between hosts and viruses, pointing to a need to reconsider current approaches aimed at finding and predicting novel zoonoses.

## Significance

Identifying whether novel human viruses disproportionately originate from certain animal groups could inform risk-based allocations of research and surveillance effort. Whether such “special reservoirs” exist remains controversial. We show that the proportion of viruses that infect humans varies minimally across reservoir taxonomic orders. Instead, the number of human-infecting viruses increases proportionately to the total number of viruses maintained by each reservoir group, which is in turn explained by the number of animal species within each group. This supports a host-neutral explanation for observed variation in the number of zoonoses among animal groups, such that traits of animal orders are unlikely to produce viruses that disproportionately threaten humans. These findings refine strategies to identify high-risk viruses prior to their emergence.

Most emerging infectious diseases of humans are viruses that originate from nonhuman animals via “zoonotic” transmission (1–3). Given the diversity of animals and viruses in nature, targeting virus discovery, surveillance, and research toward the taxonomic groups with the highest propensity to infect humans would benefit attempts to mitigate future disease outbreaks (4–7). Identifying these groups remains a major challenge. Viruses with RNA genomes are overrepresented as zoonoses and certain families including *Filoviridae*, *Orthomyxoviridae*, and *Togaviridae* contain a large proportion of zoonotic species (8, 9). Among hosts, however, patterns are less clear. Large-scale comparative studies have suggested that barriers to cross-species transmission increase with evolutionary divergence from humans, implying heightened zoonotic risk from closely related nonhuman primates (9, 10). Yet, other animal groups including bats, rodents, and ungulates are also frequently associated with zoonoses despite their evolutionary distance from humans (9, 11–13). A popular explanation (here, the “special reservoir hypothesis”) is that physiological or ecological traits of these taxa make them more likely to maintain zoonotic viruses or transmit them to humans. For example, ungulates and rodents include domesticated or anthropophilic species whose high ecological overlap with humans could facilitate pathogen exchange (13, 14). In contrast, the unique life history of bats has been hypothesized to create an evolutionarily distinct immunological environment that selects for viral traits that favor human infection (15–20). An alternative to the special reservoir hypothesis is that host species maintain a similar number of viruses with a similar per-virus risk of zoonotic transmission. Variation in the number of zoonoses among animal groups therefore arises as a consequence of their species richness (here, the “reservoir richness hypothesis”). For example, the preponderance of rodent- or bat-associated zoonoses could reflect the large number of rodent and bat species relative to other mammalian groups (21). These hypotheses imply different management strategies. The special reservoir hypothesis would advocate fundamental research to define the ecological or physiological trait profiles that explain the propensity of certain hosts to harbor zoonoses, followed by targeted surveillance or virus discovery in hosts with high-risk trait profiles. In contrast, the reservoir richness hypothesis would advocate broader surveillance and virus discovery, possibly proportionate to the local species richness of different animal groups, and would imply the need for deeper understanding of which features of virus biology enhance zoonotic transmission.

Identifying patterns in the animal origins of zoonoses has been frustrated by disconnects between the key outcome of interest, the likelihood of zoonotic transmission, and currently available data which document viral infection in human and nonhuman hosts but provide limited information on the origins of these infections. For example, humans may infect nonhuman hosts, rather than the other way around, but only the latter direction of transmission is pertinent to zoonotic origins. A greater challenge is that the reservoir host species that maintain and transmit viruses to humans can rarely be distinguished from species that form a larger community of “dead-end” hosts that are inconsequential to transmission (22, 23). Rather than reflecting genuine imbalances in the animal origins of human infections, associations between certain animal groups and zoonotic viruses based on shared detections might instead emerge from heightened surveillance for or susceptibility to zoonotic viruses in these groups even if they are not key components of natural transmission cycles. As the known diversity of viruses expands, the increasing difficulty of differentiating related viruses in different host species from the same virus transmitted between these host species would exacerbate this effect by overestimating viral sharing, particularly if relying on serological or PCR data alone (24).

Given this uncertainty in the reservoir hosts of most viruses at the species level, an alternative approach to resolving whether animal host associations influence zoonotic risk would be to assess reservoir associations at lower taxonomic resolutions. Such analyses would take advantage of the tendency for viruses to be maintained by host species within the same taxonomic order, a phenomenon which arises from more frequent host shifting among closely related species and the cospeciation of hosts and viruses (25–27). At this coarser taxonomic level, transmission cycles may be accurately defined for hundreds of viruses, enabling a test of host effects predicted by the special reservoir hypothesis (e.g., applying to animal orders such as Chiroptera or Rodentia) that is robust to reservoir species uncertainty. Further, condensing reservoir–virus associations to a single observation per virus would enable statistical approaches that separate confounding effects of virus and host taxonomy that arise from the nonrandom distribution of virus families with differing zoonotic propensity among reservoir groups. These models could also account for additional factors including differential research effort among host and virus groups and the phylogenetic proximity of animal hosts to humans that are suspected to vary with zoonotic transmission (9, 28).

## Results

**Fig. 1.**
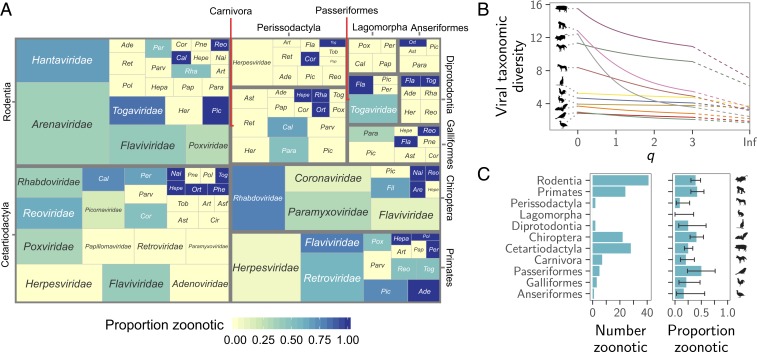
Species richness and diversity of viruses associated with major reservoir host groups. (*A*) The distribution of virus families across mammalian and avian reservoir orders. Each rectangle represents a reservoir–virus family combination, with size corresponding to the number of virus species linked to that reservoir and color indicating the proportion of these viruses which are zoonotic. Viral families are abbreviated as follows: Ade = *Adenoviridae*, Are = *Arenaviridae*, Art = *Arteriviridae*, Asf = *Asfarviridae*, Ast = *Astroviridae*, Cal = *Caliciviridae*, Cir = *Circoviridae*, Cor = *Coronaviridae*, Fil = *Filoviridae*, Fla = *Flaviviridae*, Hepa = *Hepadnaviridae*, Hepe = *Hepeviridae*, Her = *Herpesviridae*, Nai = *Nairoviridae*, Ort = *Orthomyxoviridae*, Pap = *Papillomaviridae*, Para = *Paramyxoviridae*, Parv = *Parvoviridae*, Per = *Peribunyaviridae*, Phe = *Phenuiviridae*, Pic = *Picornaviridae*, Pne = *Pneumoviridae*, Pol = *Polyomaviridae*, Pox = *Poxviridae*, Reo = *Reoviridae*, Ret = *Retroviridae*, Rha = *Rhabdoviridae*, Tob = *Tobaniviridae*, and Tog = *Togaviridae*. (*B*) The taxonomic diversity of viruses maintained by each reservoir, at decreasing levels of sensitivity to rare lineages as the *q*-parameter increases. (*C*) The number and proportion of virus species associated with each reservoir which are zoonotic; error bars show 95% binomial CIs calculated using the Wilson method (31).

We used literature searches to expand an existing dataset of the maintenance cycles of single-stranded RNA viruses (27) to include all 35 RNA and DNA virus families infecting birds and mammals, the hosts that account for the vast majority of zoonoses (8). From a total of 673 virus species, 415 species from 30 families had compelling evidence for transmission by one or more of the 11 taxonomic orders of nonhuman reservoirs (three avian, eight mammalian) which met our inclusion criteria (*Materials and Methods*). When possible, viral species linked to more than one reservoir group were subdivided into reservoir order-associated viral clades (*n* = 11 species), and otherwise excluded (*n* = 2), creating a final dataset of 429 reservoir–virus associations. Data on zoonotic status (i.e., whether a virus had been reported to transmit from nonhumans to humans) were obtained from Olival et al. (9), Woolhouse and Brierley (29), and primary literature searches. We found dramatic variation in the number and diversity of viruses across reservoir groups (Fig. 1). Cetartiodactyls and rodents were the most common reservoirs in our dataset, together accounting for half of the included viruses (50.6%), while anseriforms (waterfowl) were the most poorly represented, with only 1.4% of viruses (Fig. 1*A*). We quantified the degree of skew in viral diversity among groups of reservoirs using a sliding metric which places varying amounts of emphasis on rare viral lineages (30), here defined as those virus taxonomic lineages that were rarely found in the focal reservoir order. Despite harboring substantial viral species richness, the taxonomic distribution of viruses was notably skewed for some host groups (Fig. 1*B*). The majority of primate and rodent viruses fell in two to three viral families and the remaining viruses were scattered across families that contained few other species infecting these host groups (on average, two species; Fig. 1 *A* and *B*). Other groups including carnivores and odd-toed ungulates had smaller, but more balanced, viral communities (Fig. 1*B*). Both the number and the proportion of zoonotic virus species appeared to differ among reservoir groups, although the 95% CI of proportions overlapped in most cases (Fig. 1*C*). The taxonomic distribution of zoonoses within host groups was highly skewed (Fig. 1*A*). For example, 50% of zoonotic bat viruses were rhabdoviruses and 53.7% of zoonotic rodent viruses were hantaviruses or arenaviruses, reinforcing the need to account for virus taxonomy to avoid detecting effects of reservoirs on zoonotic status that would be unlikely to generalize across bat or rodent viruses (*SI Appendix*, Fig. S1).

**Fig. 2.**
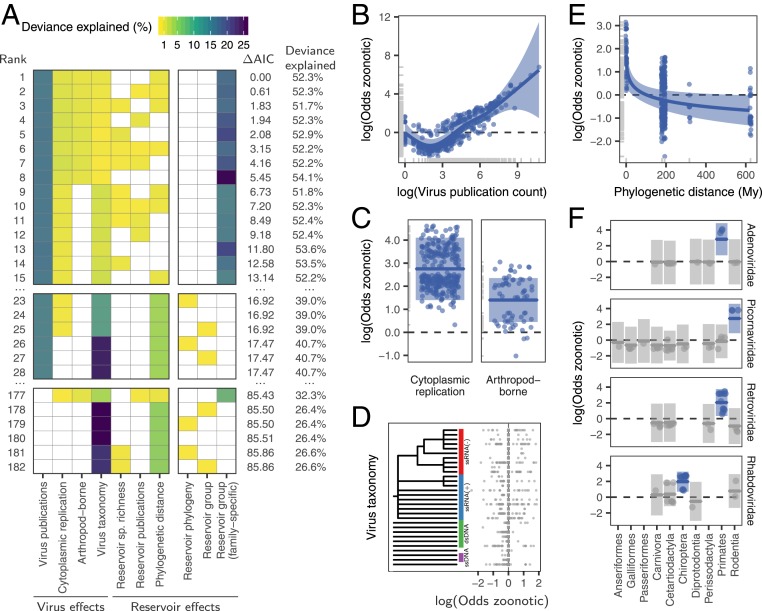
Reservoir host and virus predictors of zoonotic propensity. (*A*) Top 15 models ranked by AIC, along with the top models not containing a virus-family specific effect (ranked 23rd to 28th), and the top models not containing an effect for virus publication count (ranked 177th to 182nd). Rows represent individual models and columns represent variables. Cells are shaded according to the proportion of deviance explained by each effect; effects not present in particular models are indicated in white. The final three columns represent different versions of a potential “special reservoir effect” and were not allowed to cooccur in the same model. (*B*–*F*) Effects present in the top model. Lines indicate the predicted effect of each variable, when keeping all other variables at either their median observed value (when numeric) or their most common value (when categorical). Shaded regions indicate the 95% CIs of predictions, while points indicate partial residuals after accounting for all variables in the model except the one on the *x* axis. Effects whose 95% CI cross zero over the entire range of the predictor variable are shaded in gray. Phylogenetic distance (*E*) was measured as cophenetic distances, which describe the total evolutionary distance from each group to primates. Note that only the subset of virus families which include significant effects (i.e., those showing no overlap with 0) are illustrated in *F* (see *SI Appendix*, Fig. S2 for all families).

We next used generalized additive mixed models with zoonotic status as a binary response variable to identify and rank the host and/or viral traits that influenced the zoonotic status of viruses. When ranking models using the Akaike information criterion (AIC), the top model explained a total of 52.3% of deviance in zoonotic status (Fig. 2*A*). This model contained several previously reported effects of viral biology, including transmission by arthropod vectors and exclusively cytoplasm-based replication, which increased the odds of being zoonotic 4.1-fold (log odds = 1.40) and 15.7-fold (log odds = 2.75), respectively (Fig. 2*C*). However, neither explained >2.2% of the deviance (Fig. 2*A*). Zoonotic viruses also tended to be more studied, with the number of publications associated with each virus species explaining 15.2% of the deviance (Fig. 2*B*).

Among effects related to reservoir hosts, zoonotic risk declined with greater phylogenetic distance from reservoirs to primates, but this effect explained only 0.7% of the deviance in the top model and was absent from other competitive models (Fig. 2 *A* and *E*). Effects of reservoir order or phylogeny (either alone or crossed with an effect of virus family-level taxonomy) did not occur until the 23rd-ranked model, where they explained <0.01% of the deviance (ΔAIC from top model >16.9; Fig. 2*A*). The only strongly supported models containing an effect of reservoir host restrained the effect to certain viral families (i.e., a random effect of reservoir nested within virus family, here termed the “virus family-specific reservoir” effect). This effect explained 20.7% of the deviance in zoonotic status in the top model. In particular, primate adenoviruses and retroviruses, bat rhabdoviruses, and rodent picornaviruses were more likely to be zoonotic relative to viruses from the same families associated with other reservoirs (Fig. 2*F*). Critically however, none of these reservoir groups increased zoonotic propensity across additional viral families, suggesting the absence of generalizable effects of reservoir group on the likelihood of zoonotic transmission (Fig. 2*A* and *SI Appendix*, Fig. S2).

We further evaluated whether effects of reservoir hosts on zoonotic status might have been missed due to the inclusion of potentially collinear variables or insufficient statistical power to fit complex models. First, given that study effort understandably increased for zoonotic viruses, including this factor might have disguised true reservoir effects. However, models which did not adjust for virus-related publication counts failed to support reservoir effects; the best of such models achieved similar AIC values with and without reservoir effects (models 177 to 182, Fig. 2*A*). Second, including virus taxonomy might have reduced the power of our models. However, reservoir effects remained negligible or absent in models which excluded viral taxonomy (*SI Appendix*, Fig. S3). In contrast, viral family effects were robust to inclusion of general reservoir effects (i.e., those testing for consistent reservoir effects across viral families). When the family-specific reservoir effect was not present, viral rather than reservoir taxonomy was the stronger predictor of zoonotic status (models 23 to 28 in Fig. 2*A* and *SI Appendix*, Fig. S3). Thus, our results provide no support for the special reservoir hypothesis, which predicted that viruses associated with certain reservoir groups would be more likely to be zoonotic.

**Fig. 3.**
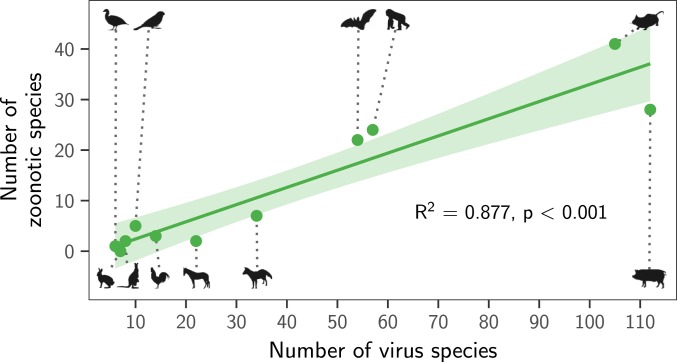
Relationship between the number of virus species and the number of zoonotic species maintained by each reservoir group. The line shows a linear regression fit, with its 95% CI indicated by the shading.

**Fig. 4.**
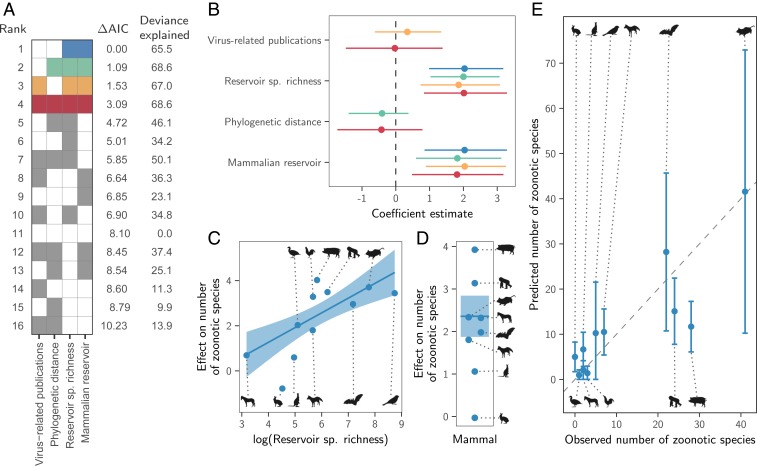
Factors predicting the number of zoonotic virus species across animal orders. (*A*) Models for all possible variable combinations ranked by AIC. Each row represents a model, while columns represent variables. Filled cells and white cells indicate variable inclusion and absence, respectively. The top four model are color-coded, with colors reused in all other panels to identify the respective models. (*B*) Coefficient estimates for the top four models; points indicate the maximum likelihood estimate and lines show 95% CIs. All variables were scaled by dividing them by 2 times their SD, meaning coefficients are directly comparable as effect sizes. (*C* and *D*) Partial effect plots for variables in the top model. Lines and shading indicate the partial effects and 95% CIs, with points showing partial residuals. (*E*) Predicted number of zoonotic viruses for each reservoir group when using the top model (blue in *A*; see *SI Appendix*, Fig. S5 for other top models).

Instead, both the total number of viruses associated with each reservoir group (“viral richness”) and the number of zoonoses matched expectations under the reservoir richness hypothesis. The absence of reservoir effects meant that viral richness was strongly correlated with the number of zoonotic species (*R*^2^ = 0.88, *P* < 0.001; Fig. 3), consistent with a similar per-virus zoonotic risk across reservoirs. Notably, a similar correlation was previously observed when measuring viral richness at the host species level (9). A series of negative-binomial generalized linear models showed that—after controlling for greater viral richness in mammals compared to birds—reservoir species richness was correlated with both the number of zoonotic viruses (65.5% of deviance) and the viral richness (56.4% of deviance) of different reservoir orders (Fig. 4 and *SI Appendix*, Fig. S4). These models outcompeted those including previously identified effects of virus-related research effort and the phylogenetic distance of each reservoir group from primates (9), which when included explained a maximum of 3.1% additional deviance (ΔAIC ≥ 1.09; models 2 to 4 in Fig. 4*A*). Exchanging the reservoir class effect (i.e., birds versus mammals) for phylogenetic distance to primates performed considerably worse than the top model (ΔAIC = 4.72; model 5 in Fig. 4*A*). Similar results were observed for models predicting total viral richness (*SI Appendix*, Fig. S4).

We next assessed how well the predictions of the reservoir richness model matched observations in individual reservoir groups. Highly recognized mammalian reservoir groups including bats and rodents hosted close to the number of zoonoses expected from their species richness (bats: 22 observed and 28 predicted [95% CI: 10.7 to 45.7]; rodents: 41 observed and 42 predicted [95% CI: 10.2 to 72.9]; Fig. 4*E*). In contrast, lagomorphs (rabbits, hares, and pikas) and diprotodonts (an order of marsupials) hosted both fewer viruses and fewer zoonoses than predicted; however, these were among the least-studied animal groups (Fig. 4*E* and *SI Appendix*, Fig. S4*E*). The only potentially “special” reservoirs identified by this analysis were the cetartiodactyls (even-toed ungulates and whales) and primates, which hosted more zoonoses than would be predicted for mammalian groups with their species richness (Fig. 4*E*). In the case of primates, this likely reflects the higher zoonotic propensity of primate-borne adeno- and retroviruses identified above (24 zoonotic viruses observed, of which 3 are adenoviruses and 7 are retroviruses; 15 predicted [95% CI: 7.8 to 22.4]). The mismatch was greater for cetartiodactyls (28 observed, 12 predicted [95% CI: 6.1 to 17.3]). However, perhaps due to their close association with humans as domestic livestock, this group also had considerably higher total viral richness than predicted (112 observed and 45 predicted [95% CI: 25.3 to 65.5]; *SI Appendix*, Fig. S4*E*). Consequently, the proportion of cetartiodactyl viruses which were zoonotic was unexceptional, inconsistent with the special reservoir hypothesis (Figs. 1 and 3). Strikingly, although mammals had elevated viral and zoonosis richness compared to birds, the proportion of zoonotic viruses was similar between classes, with 9/30 of avian and 115/387 of mammalian virus species being zoonotic (30% and 28.8%, respectively), emphasizing that—regardless of the cause—it is the underlying number of virus species which differs between mammals and birds, not the zoonotic component.

## Discussion

Animal groups have been proposed to differ in their propensity to transmit viruses to humans as a consequence of variation in their life history, physiology, or ecology. By combining a large dataset of reservoir host–virus associations with records describing their histories of human infection, our analysis found no evidence that the taxonomic identity of reservoirs affects the probability that the viruses they harbor are zoonotic. Instead, variation in the number of zoonoses maintained by each reservoir group was consistent with a largely host-neutral model, whereby more species-rich reservoir groups host more virus species and therefore a larger number of zoonotic species. These findings imply the need to reconsider some current approaches to virus discovery, surveillance, and research.

The absence of effects of reservoir host associations on the zoonotic status of viruses is at odds with the idea that conserved traits of certain host groups alter the zoonotic potential of their viruses. If host traits predictably altered zoonotic risk, these effects would be expected to act on multiple viral groups. Instead, we found that reservoir effects were isolated within individual virus families, such that no reservoir group altered the zoonotic risk of viruses across a broad range of viral families. For example, bats are widely considered special reservoirs due to their association with several high-profile zoonoses, including *Severe acute respiratory syndrome-related coronavirus* (*Coronaviridae*), *Nipah henipavirus* (*Paramyxoviridae*), and Ebola viruses (*Filoviridae*) (17, 19, 31). However, virus species within those families with a bat reservoir were no more likely to be zoonotic than those transmitted by other hosts, although we acknowledge that this effect would have been difficult to detect for filoviruses given the small number of virus species in this family. While our results do not dispute the existence of distinct features of bat immunity or life history which have been hypothesized to influence viral communities in bats (16, 18, 19, 32, 33), they provide no compelling evidence that these traits translate into an increased probability of bat-associated viruses infecting humans.

Idiosyncratic family-specific reservoir effects could reflect especially zoonotic clades within viral families that are strongly host-associated, sampling biases, or genuine interactions between the biological features of specific viral groups and specific reservoirs that increase zoonotic capability (e.g., differences in viral shedding or tissue tropism). Among rhabdoviruses, a significantly elevated proportion of bat-associated species were zoonotic, but this effect was driven by the rabies-causing genus *Lyssavirus*, which contained 73% of the 25 zoonotic rhabdovirus species and is strongly bat-associated (35). However, *Rabies lyssavirus* is also zoonotic when associated with carnivores and bat-associated Rhabdoviruses outside of the Lyssaviruses are not known to be zoonotic, suggesting that traits of this viral genus rather than its reservoirs are the underlying driver of the detected bat effect (*SI Appendix*, Fig. S3). The putatively heightened frequency of zoonotic rodent-associated picornaviruses might conceivably arise if transmission by rodents selects for viral traits that disproportionately promote zoonotic capability or if picornaviruses have similar zoonotic capability across reservoir groups but heightened ecological overlap favors zoonotic transmission from rodents. Yet, given that our dataset contained only three rodent-associated picornaviruses (all zoonotic) and primates and cetartiodactyls also hosted zoonotic picornaviruses (three out of four and one out of six zoonotic, respectively), a statistical artifact of low sample size cannot be ruled out. In contrast, effects of nonhuman primate reservoirs on the zoonotic potential of adenoviruses and retroviruses may have a biological basis. All primate-associated adenoviruses (three out of three) and 7 out of 12 primate-associated retroviruses were zoonotic, while none of the 15 adenoviruses and 16 retroviruses associated with other reservoirs have been reported to infect humans. This suggests that evolutionarily conserved similarities between nonhuman primates and humans may facilitate zoonotic transmission in these viral families. Yet, our results also highlight limitations of host phylogeny as a generalizable predictor of zoonotic capability since primate associations did not affect the zoonotic status in most of the viral families maintained by nonhuman primates. We hypothesize that the advantages conferred by phylogenetic relatedness are crucial for viral families that are inherently host-restricted (e.g., retroviruses, which must integrate into host genomes) but are less influential for viruses with less specialized infection and replication mechanisms. More generally, the relatively poor performance of phylogenetic distance in predicting either the proportion or the number of zoonoses from different reservoir groups (Figs. 2*E* and 4 *A* and *B*) suggests that the evolutionarily conserved factors that facilitate cross-species transmission within animal orders (25, 36, 37) are unlikely to extend over deeper evolutionary distances.

Our modeling framework allowed us to distinguish the relative contribution of host and virus features to zoonotic status. Consistent effects of viral taxonomy support the conclusion that zoonotic ability is a feature of viral clades rather than host associations (9, 28); however, the underlying viral trait determinants remain unresolved. As shown here (Fig. 2) and elsewhere, transmission by arthropod vectors and replication in the cytoplasm of host cells were linked to zoonotic transmission (9, 38). These findings are potentially explainable by selection for viral generalism arising from the need to replicate in both vertebrate and invertebrate hosts and the avoidance of the additional barriers associated with entering and replicating in the nucleus in novel host species, respectively. Yet, the site of viral replication within cells is invariant within viral families and arthropod-borne transmission is also strongly evolutionarily conserved (27, 39). Therefore, these traits have limited utility for explaining large variation in zoonotic risk within viral families. Identifying whether factors beyond ecological opportunity predict zoonotic status is vitally important to anticipate zoonotic transmission and to estimate the number of viruses which may emerge in the future.

The number of zoonoses scaled positively with the host species richness of animal orders (Fig. 4). Since reservoir species richness did not affect the probability that viruses were zoonotic (Fig. 2*A*), direct effects of species richness—which might occur if viral maintenance by more species rich host orders facilitates zoonotic transmission by selecting for broader host range—are unlikely (2). Instead, our results support a numerical, host species-neutral explanation: more species-rich animal orders maintain more viruses and hence more zoonoses. Indeed, after controlling for diminished viral richness in birds compared to mammals, key groups including bats and rodents harbored close to the number of zoonoses expected from their species richness (Fig. 4). These results mirror—and potentially explain—observations that the risk of zoonotic emergence is elevated in regions with high species richness (3, 40), since more virus species and hence zoonoses would be expected in species-rich habitats. More generally, our results suggest that the processes which shape viral richness (i.e., extinction, codivergence, and host shifts) do not vary enough among animal orders to create significant differences in the average number of viruses per species across reservoir orders. One implication of the relationship between reservoir species richness and the number of zoonoses is that surveillance efforts aimed at finding potential zoonoses should scale with the species richness of reservoir groups. This recommendation differs from the current practice which is based on the a priori expectation that certain groups (e.g., bats, rodents, and primates) are more likely to maintain zoonotic viruses (7). Analogously, our results underscore the challenges of identifying unknown reservoirs for viruses of importance to human health, since sampling effort would need to scale with local biodiversity, which itself may be uncertain.

Our dataset and analytical approach differed in several epidemiologically important ways from previous studies which suggested variation in viral zoonotic propensity across animal groups (9, 12). First, restricting virus–reservoir associations to the order level allowed us to test hypotheses on the role of broad animal groups on zoonotic origins in the face of widespread uncertainty in the identity of viral reservoirs at the host species level (22, 23, 27). This meant excluding human viruses that occasionally infect nonhumans and all associations between viruses and nonhuman hosts which are not currently considered to be important in natural transmission cycles. While it is conceivable that some excluded host–virus associations have an unrecognized role in transmission, adding 302 previously reported associations which did not meet our criteria did not qualitatively change our results (*SI Appendix*, Fig. S6). Second, we considered a single reservoir order for most viruses rather than modeling every association of each virus with each infected host species within that order independently. This was critical to avoid potentially spurious effects driven by surveillance effort and multiple observations of the same virus in closely related species. For example, *Dengue virus* (DENV) has been detected in at least 20 bat species, which comprise 76% of DENV–host associations (9, 12), but nonhuman primates, not bats, are the currently accepted reservoirs of zoonotic DENV outbreaks (41–43). Our dataset therefore included a single primate reservoir since the majority of DENV–host associations would have obscured conclusions on zoonotic origins. Finally, unlike previous studies, we included avian viruses; however, restricting our analysis to mammalian viruses failed to recover reservoir host effects on zoonotic risk (*SI Appendix*, Fig. S7). One possible explanation for the apparent discrepancy between our findings and those based on shared virus detections could be that heightened surveillance for or susceptibility to zoonoses has led to elevated detections of spillover infections in previously identified “special hosts.” Our results highlight the importance of separating exposure and infection from transmission in future studies which involve reservoirs of infection.

A clear limitation of our approach was that we were unable to consider traits that vary within reservoir groups (e.g., reproductive rates, population size, and geographic range size) which may moderate the baseline zoonotic risk by altering viral richness, transmission dynamics, or ecological contacts of hosts with humans (44). In addition, even at the broad level of host taxonomy we used, knowledge of virus–reservoir relationships and zoonotic capability is incomplete and unevenly distributed among host groups (45). Analogous analyses of the capability of viruses to infect nonhuman hosts could also enable broader understanding of the determinants of cross-species transmission but will require constructing comprehensive datasets of infection histories in these alternative species. Finally, we evaluated only viral richness and whether viruses were reported to infect humans. Whether viruses from different reservoirs differ systematically in their pathogenicity, capacity to transmit among humans, or in their frequency of zoonotic transmission cannot be assessed from our data.

In summary, our analysis suggests that variation in the frequency of zoonoses among major bird and mammal reservoir groups is an emergent property of variation in host and virus species richness. We find no evidence that intrinsic or ecological differences among animal groups increases the number of viruses they maintain or the likelihood that any given virus is zoonotic. Basing public health surveillance and research strategies aiming to identify high-risk viruses on the assumption that some taxonomic orders of hosts are disproportionate sources of zoonoses risks missing important zoonotic viruses while simultaneously reenforcing patterns that may reflect detection biases rather than zoonotic risk.

## Materials and Methods

### Database Construction.

We studied 35 virus families listed as infecting animals in either Fields Virology or the ViralZone web resource (*Adenoviridae*, *Anelloviridae*, *Arenaviridae*, *Arteriviridae*, *Asfarviridae*, *Astroviridae*, *Birnaviridae*, *Bornaviridae*, *Caliciviridae*, *Circoviridae*, *Coronaviridae*, *Filoviridae*, *Flaviviridae*, *Genomoviridae*, *Hantaviridae*, *Hepadnaviridae*, *Hepeviridae*, *Herpesviridae*, *Nairoviridae*, *Orthomyxoviridae*, *Papillomaviridae*, *Paramyxoviridae*, *Parvoviridae*, *Peribunyaviridae*, *Phenuiviridae*, *Picobirnaviridae*, *Picornaviridae*, *Pneumoviridae*, *Polyomaviridae*, *Poxviridae*, *Reoviridae*, *Retroviridae*, *Rhabdoviridae*, *Tobaniviridae*, and *Togaviridae*) (39, 46). For all data-collection steps below, virus names were matched to the latest accepted taxonomy according to version 2018b of the ICTV Master Species List by referring to historical taxonomic releases (47).

Following Babayan et al. (27), data on the reservoirs known to maintain the included virus species were obtained by searching common virology textbooks, such as *Fields Virology* (39), followed by in-depth literature searches until at least one reservoir or consistent evidence that the reservoir is currently considered as unknown was found. We summarized viral maintenance to the level of taxonomic orders of reservoirs because 1) the special reservoir hypothesis is generally articulated in terms of taxonomic order (e.g., applying to Chiroptera or Rodentia) and 2) order-level analyses constituted a reasonable compromise between taxonomic resolution and sample size, maximizing the number of virus species which could be included (27). We supplemented the single-stranded RNA virus dataset of Babayan et al. (27) with 344 additional records representing all recognized species in the families above for which reservoirs could be found. When there was evidence for independent maintenance by multiple reservoir orders, all known virus species–reservoir order combinations were recorded. For example, *R*abies* lyssavirus* is maintained by both Carnivora and Chiroptera, but never as part of the same transmission cycle (48). Nineteen such virus species were detected, associated with 2.5 reservoir orders on average. Viruses known to be maintained by humans were retained if there was evidence for independent maintenance of a separate viral lineage in nonhuman hosts (*n* = 25) and otherwise discarded (*n* = 72). *Yellow fever virus*, for example, can be maintained by humans but also has an independent nonhuman primate reservoir community, and lineages from such maintenance cycles are zoonotic (39). Two virus species, *Mammalian orthoreovirus* and *Usutu virus*, had reservoir communities currently thought to span multiple taxonomic orders and were excluded from further analysis. Finally, the dataset was restricted to contain only viruses associated with mammalian and avian reservoir groups for which at least five viruses were known. The zoonotic status of individual virus species was obtained by combining records of detected human infections from Olival et al. (9) (*n* = 342 virus species) and Woolhouse and Brierley (29) (*n* = 70) with additional literature searching (*n* = 3). Only detections where the identity of the virus infecting humans was confirmed to species level by PCR or sequencing were considered.

### Statistical Modeling.

We used logistic regression models to assess the association between reservoir group and zoonotic status (a binary response variable). Because at least one previously reported predictor of zoonotic status—publication count—was expected to be nonlinear (9), we used generalized additive mixed models (48). Three possible representations of a reservoir host effect were included (described below in more detail) and combined with all possible combinations of additional variables that were previously reported to predict zoonotic status. These included measures of research effort, the phylogenetic distance between each reservoir taxonomic order and primates, the species richness of each reservoir order, whether or not the virus was transmitted by arthropods or replicated exclusively in the cytoplasm, and a hierarchical representation of virus taxonomy. Previously reported variables relating to host species, such as geographic overlap with humans, could not be included when summarizing reservoirs at the level of taxonomic order.

The simplest reservoir effect was a random intercept for each reservoir taxonomic order, with all reservoir orders assumed independent. This represented the typically assumed special reservoir effect, in which some reservoirs are more prone to maintain zoonotic viruses than others. A second representation allowed clustering on the reservoir phylogeny to represent the hypothesis that related reservoir groups have correlated associations with zoonotic viruses. For example, mammalian reservoirs may be associated with a larger fraction of zoonoses than avian reservoirs, or related mammalian clades may share phylogenetically conserved features which shape their association with zoonoses. This phylogenetic random effect was implemented as a multivariate normal distribution, where phylogenetic relationships among taxa determined the amount of correlation between randomly sampled intercepts for different reservoir taxonomic orders (51). The variance–covariance matrix of this distribution took the form $\sigma^{2}A$, where $\sigma^{2}$ is a variance parameter to be estimated and A is a known variance–covariance matrix derived from a phylogeny (51, 52). We used a composite time-scaled reservoir phylogeny representing the mean divergence dates for all clades estimated across multiple studies, as contained in the TimeTree database (51). Following Longdon et al. (36), this phylogeny was converted to a variance–covariance matrix by assuming a Brownian motion model of trait evolution, using the vcv.phylo function in the APE package in R version 3.5.1 (52, 53). The third representation of a reservoir effect allowed independent random intercepts for each combination of reservoir order and virus family (i.e., the random effect of reservoir order was nested within virus family). This represented the hypothesis that specific reservoirs influence the propensity for viruses to be zoonotic for only some virus families, or that the identity of special reservoirs differs between families.

We used two measures to correct for the effects of variation in research effort on observations of zoonotic transmission: the numbers of results matching PubMed Central queries related to 1) virus species and 2) reservoir group (on 1 November 2019). For each virus species, this query was “<taxid>[Organism:noexp],” where “<taxid>” was replaced by the NCBI Taxonomy ID corresponding to each virus species. Viruses with no entry in the NCBI taxonomy database were removed from all analyses (this affected *Cercopithecine gammaherpesvirus 14* and *Hare fibroma virus*, neither of which have publicly available sequence data in GenBank, meaning their reported reservoir associations are questionable). To capture a measure of the virus-related research effort in each reservoir group, a set of queries of the form “<taxid>[Organism:noexp] AND virus” were constructed, replacing “<taxid>” with the NCBI Taxonomy ID of each taxonomic order in turn. For nonhuman primates, this query was modified to “(txid9443[Organism:noexp] NOT txid9606[Organism:noexp]) AND virus,” where “txid9443” is the Taxonomy ID for the order Primates, and “txid9606” is the Taxonomy ID specific to *Homo sapiens*. Both variables were included as the natural logarithm of the respective publication counts, reflecting our prior belief that the effect of increasing numbers of publications would become saturated at high values. Nevertheless, we also allowed for additional nonlinear effects by fitting both variables as thin-plate smooths with 10 and 8 knots for virus and reservoir publication counts, respectively (54).

Phylogenetic distance was calculated as the cophenetic distance between each reservoir order and primates, using the same time-scaled reservoir phylogeny as above. Following Olival et al. (9), this effect was log(x + 1)-transformed in all models. The species richness represented by each reservoir order was derived from the Catalogue of Life using version 0.9.6 of the taxize library in R (55, 56). Both variables were fit as a thin-plate smooths with 6 and 10 knots, respectively.

The taxonomic random effect of virus family used the same specification as the reservoir phylogenetic effect above. Because the included virus families were too diverse to be represented in a single phylogeny (and might not share a common ancestor), a variance–covariance matrix representing currently accepted virus taxonomic relationships was generated. This matrix reflects the proportion of the taxonomy that is shared between virus families. Specifically, pairs of viruses were assigned a similarity score N/max(N), where N is the number of taxonomic levels they share, and max(N) the total number of taxonomic levels. This calculation was performed considering all taxonomic levels from realm to family. Thus, viruses in the same virus family received a similarity value of 1, while viruses from different families in the same order (e.g., *Bunyavirales*) received a value of 0.8. Virus species sharing no taxonomic levels were treated as independent (similarity = 0). Missing taxonomic assignments were interpolated to retain a comparable number of levels across all viruses, ensuring that all viruses remained independent at all levels not supported by the official ICTV taxonomy. For example, since many viruses are not classified using suborders a gap between order and family was bridged by creating a new level, assigning each family to its own unique suborder. This approach generated several free-floating branches, which are assumed independent in our models, but this is consistent with current virus taxonomy (ICTV Master Species List 2018b, https://ictv.global/files/master-species-lists/; *SI Appendix*, Fig. S3).

All models were fit using restricted maximum likelihood in the mgcv library in R and subsequently ranked by AIC (57, 58). The validity of models was checked using both standard methods implemented in the mgcv library and by inspecting simulated residuals generated using the DHARMa library in R (59). To calculate the proportion of the deviance explained by each term, each model was compared to submodels fit in the absence of individual terms, fixing the values for the smoothing parameters of the remaining terms to those estimated in the full model. The proportion of the deviance explained was calculated as $\left(D_{i}-D_{F}\right)/D_{0}$, where $D_{i}$ is the deviance of model i, $D_{F}$ is the deviance of the full model, and $D_{0}$ is the deviance of an intercept-only model.

To investigate potential explanations for differences in either the number of virus species or the number of zoonotic species associated with each reservoir order, two independent sets of negative binomial generalized linear models (GLMs) were fit to the counts of each virus type using the MASS library in R (60). Both sets of models contained all possible combinations of variables describing the log number virus-related publications associated with each reservoir, log phylogenetic distance to primates, log species richness in each reservoir, and a binary variable describing whether the reservoir is mammalian. We considered simpler Poisson GLMs instead of the negative binomial GLMs, but these showed strong overdispersion in simulated residuals using the DHARMa library.

### Diversity Calculations.

The distribution of viral diversity among reservoir groups was quantified by calculating profiles of normalized alpha diversity using the rdiversity library in R (30). These calculations incorporated a species-level taxonomic similarity matrix, reflecting the proportion of the taxonomy that is shared between virus species and calculated as described for the family-level similarity matrix above.

### Data Availability.

Data and code used in this manuscript are available at https://doi.org/10.5281/zenodo.3516613.

## Supplementary Material

Supplementary File
