# Supplementary material for: Viral zoonotic risk is homogenous among taxonomic orders of mammalian and avian reservoir hosts
Source: Proc Natl Acad Sci U S A. 2020 Apr 13;117(17):9423–30. doi: 10.1073/pnas.1919176117 (PMC7196766; doi:10.1073/pnas.1919176117)
Supplement: Supplementary File [file pnas.1919176117.sapp.pdf]

PNAS
www.pnas.org

Supplementary Information for

Viral zoonotic risk is homogenous among taxonomic orders of mammalian and avian reservoir hosts

Nardus Mollentze and Daniel G. Streicker

E-mail: nardus.mollentze@glasgow.ac.uk / daniel.streicker@glasgow.ac.uk

This PDF file includes:

Figs. S1 to S7
SI References

Other supplementary materials for this manuscript include the following:

Data and all code (available at https://doi.org/10.5281/zenodo.3516613)

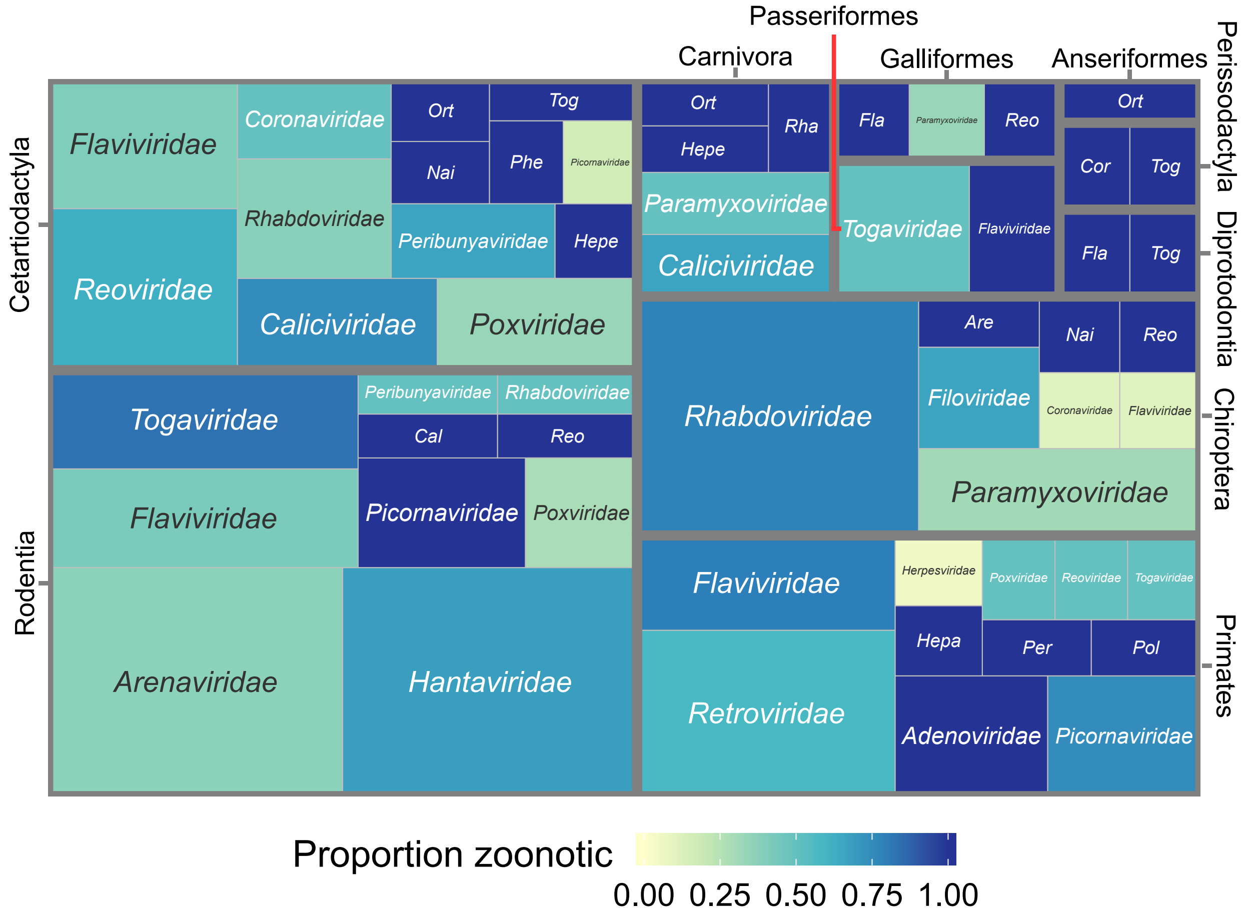

Fig. S1. Number of zoonotic viruses linked to key mammalian and avian reservoir orders. Each rectangle represents a reservoir – virus family combination, with its size corresponding to the number of zoonotic virus species definitively linked to that reservoir and its colour indicating the proportion of the total number of viruses in that reservoir–family combination that this represents. Ade = Adenoviridae, Are = Arenaviridae, Art = Arteriviridae, Asf = Asfarviridae, Ast = Astroviridae, Cal = Caliciviridae, Cor = Coronaviridae, Fla = Flaviviridae, Hepa = Hepadnaviridae, Hepe = Hepeviridae, Nai = Nairoviridae, Ort = Orthomyxoviridae, Pap = Papillomaviridae, Per = Peribunyaviridae, Phe = Phenuiviridae, Pic = Picornaviridae, Pne = Pneumoviridae, Pol = Polyomaviridae, Pox = Poxviridae, Reo = Reoviridae, Rha = Rhabdoviridae, Tob = Tobaniviridae, Tog = Togaviridae.

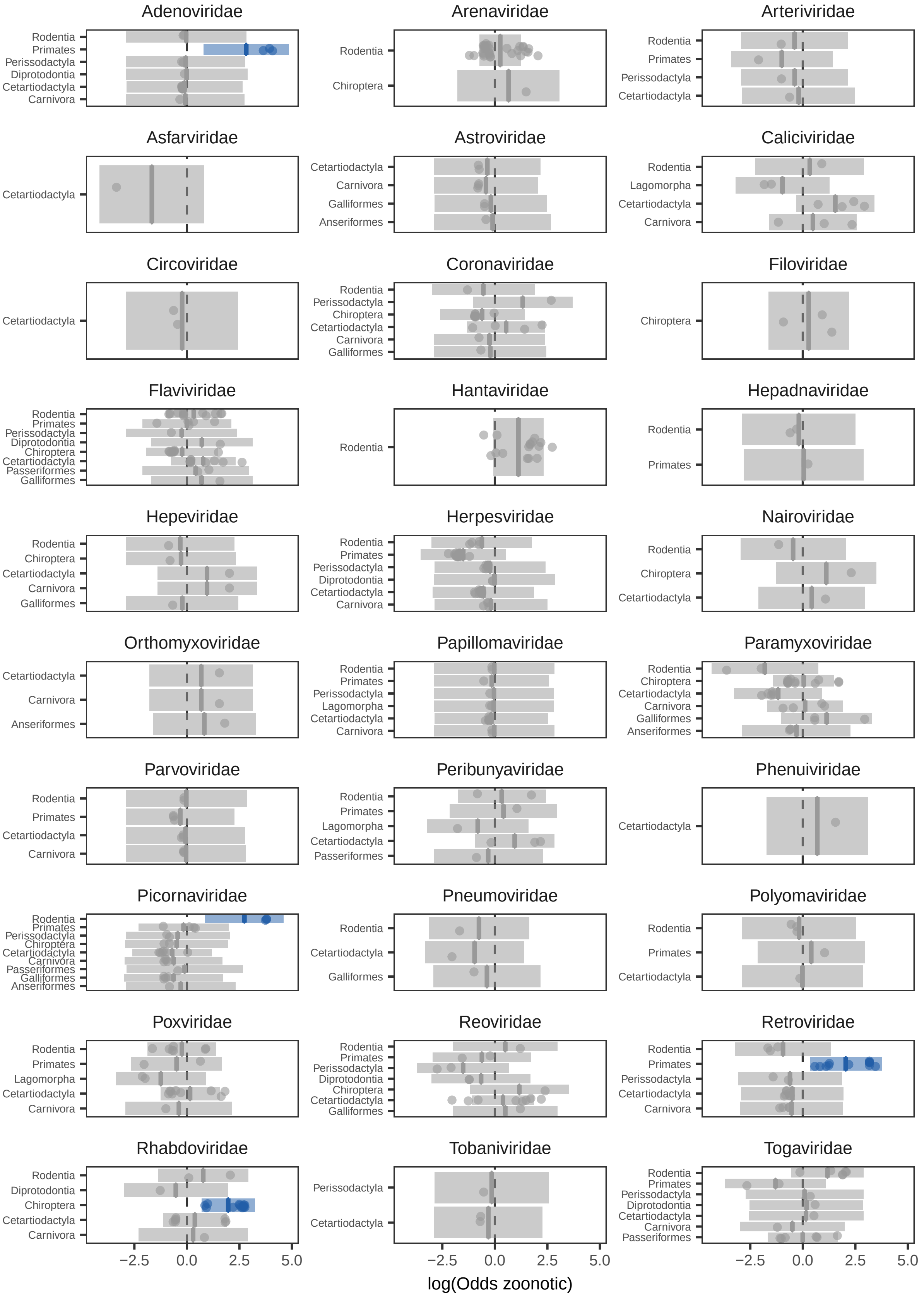

Fig. S2. Partial effects plot for the family-specific reservoir effect in the top-ranked model in Fig. 2A. Points represent partial residuals, after accounting for the other effects in the model, while lines indicate the predicted effect. Shaded areas show the 95% confidence intervals of each predicted effect. Effects which are clearly separated from 0 (defined as having a confidence interval which does not cross 0) are highlighted in blue.

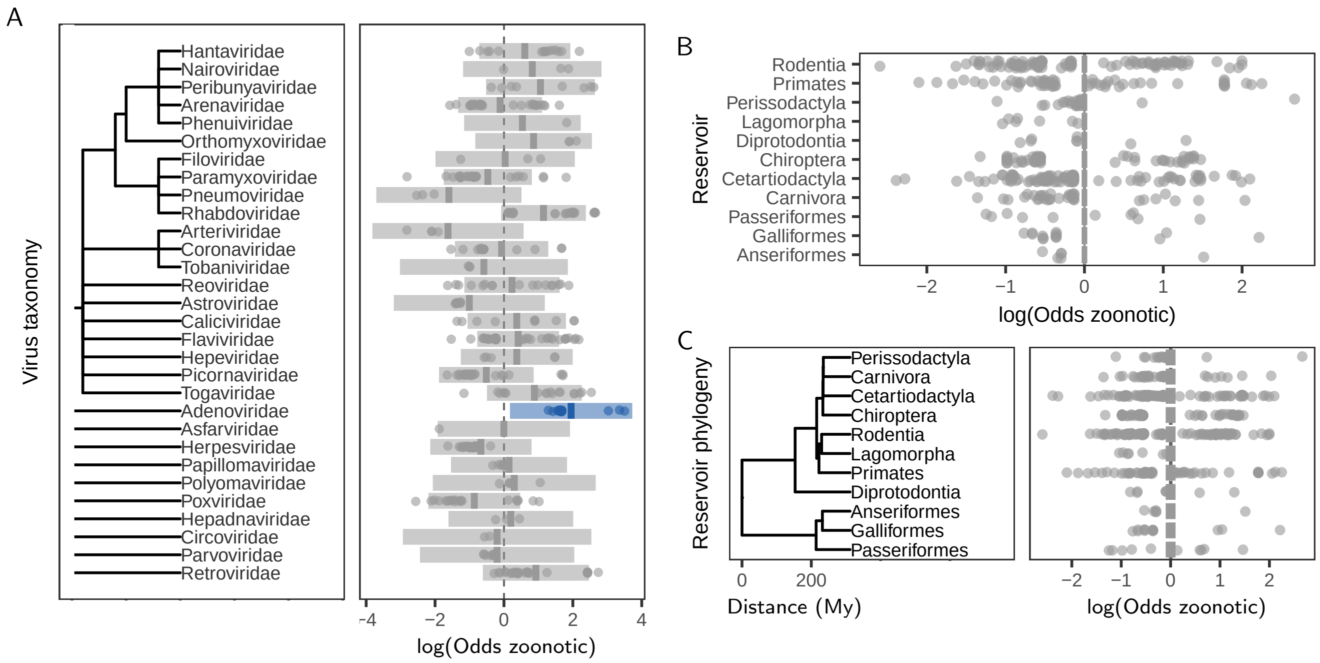

Fig. S3. Effect of virus taxonomy and reservoir phylogeny on the probability of being zoonotic, after accounting for all other effects in the model. (A) Partial effects for the individual families making up the taxonomic random effect, in the best model containing a virus taxonomy effect in the absence of any reservoir-related random effects (ranked 34th in Fig. 2; ΔAIC = 14.1). (B-C) Partial effects for the reservoir and reservoir phylogeny random effects from the top-ranked model in which each occurred without a virus taxonomy random effect (ranked 127th and 128th, respectively; ΔAIC = 30.8 in both cases). Bars indicate partial effects, after accounting for all other variables in the model, with shaded regions showing the extent of their 95% confidence intervals, while points indicate partial residuals for individual virus species. Families with effects clearly separated from 0 are highlighted in blue.

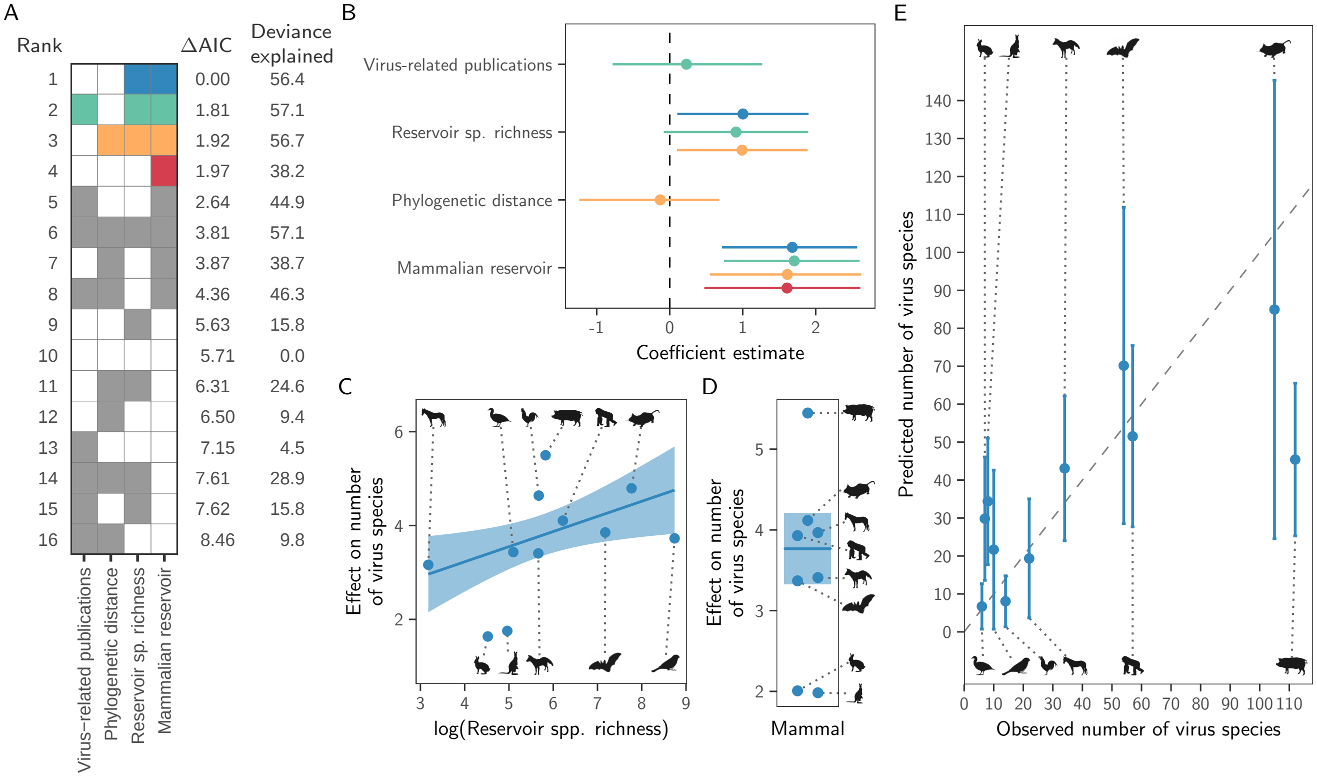

Rank	ΔAIC	Deviance explained
1	0.00	56.4
2	1.81	57.1
3	1.92	56.7
4	1.97	38.2
5	2.64	44.9
6	3.81	57.1
7	3.87	38.7
8	4.36	46.3
9	5.63	15.8
10	5.71	0.0
11	6.31	24.6
12	6.50	9.4
13	7.15	4.5
14	7.61	28.9
15	7.62	15.8
16	8.46	9.8

Fig. S4. Factors predicting the total number virus species maintained by different animal orders (viral richness). (A) Models for all possible variable combinations ranked by AIC. Each row represents a model, while columns represent variables. Filled cells and white cells indicate variable inclusion and absence, respectively. The top four model are colour coded, with colours re-used in all other panels to identify the respective models. (B) Coefficient estimates for the top 4 models: points indicate the maximum likelihood estimate; lines show 95% confidence intervals. All variables were scaled by dividing them by 2 times their standard deviation. (C–D) Partial effect plots for variables in the top model. Lines and shading indicate the partial effects and 95% confidence intervals, with points showing partial residuals. (E) Predicted viral richness for each reservoir group when using the top model (blue in panel A).

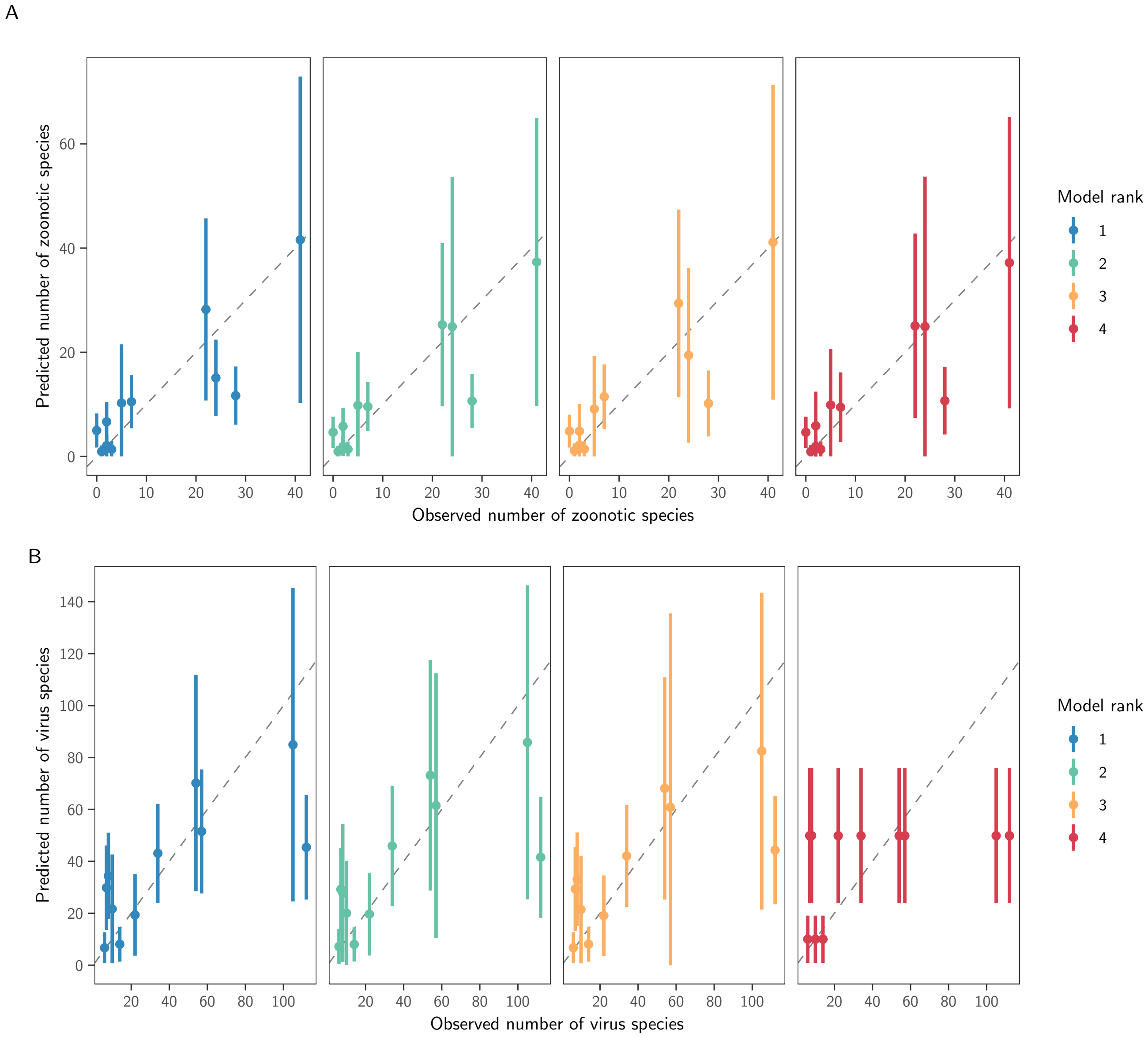

Fig. S5. Predictions from the top four models predicting the number of zoonotic species and the total viral richness. (A) Observed and predicted number of zoonotic species for each reservoir group when using each of the top four models in Fig. 4A. (B) Observed and predicted total viral richness per reservoir group, for the top four models illustrated in Fig. S4A.

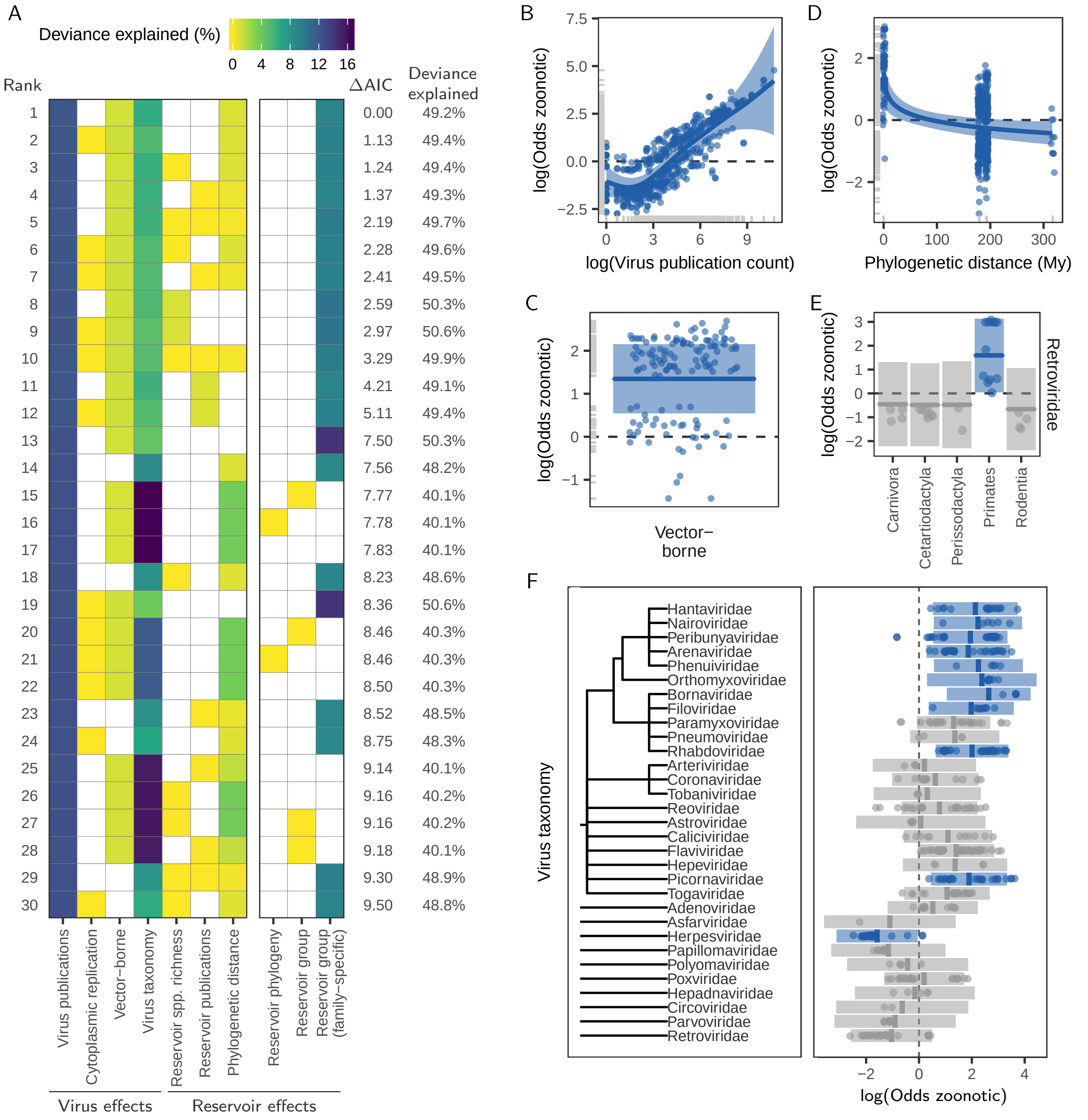

Fig. S6. Predictors of zoonotic propensity when including hosts in which viruses have been detected, but for which maintenance has not been confirmed. After updating virus names to the most recent ICTV-approved taxonomy (Master Species List 2018b, https://ictv.global/files/master-species-lists/), PCR-confirmed virus detections from (1) where summarised to the host taxonomic order level, retaining unique combinations of virus species with each of the reservoir orders included in the primary analysis. These host-virus interactions were added to the list of known reservoir-virus interactions before repeating the analyses described in the main text. Because the dataset of (1) did not contain avian hosts, these were removed from the reservoir data, although results were similar when including them. The top 30 models ranked by AIC are shown in (A), with rows representing individual models and columns representing variables. The effects present in the top model are illustrated in panels B – F, with lines indicating the predicted effect of the variable on the x-axis, when keeping all other variables at their mean observed value, and shaded regions indicating the 95% confidence interval of this prediction. Points illustrate partial residuals. Effects whose 95% confidence interval cross 0 over the entire range of the predictor variable are shaded in grey.

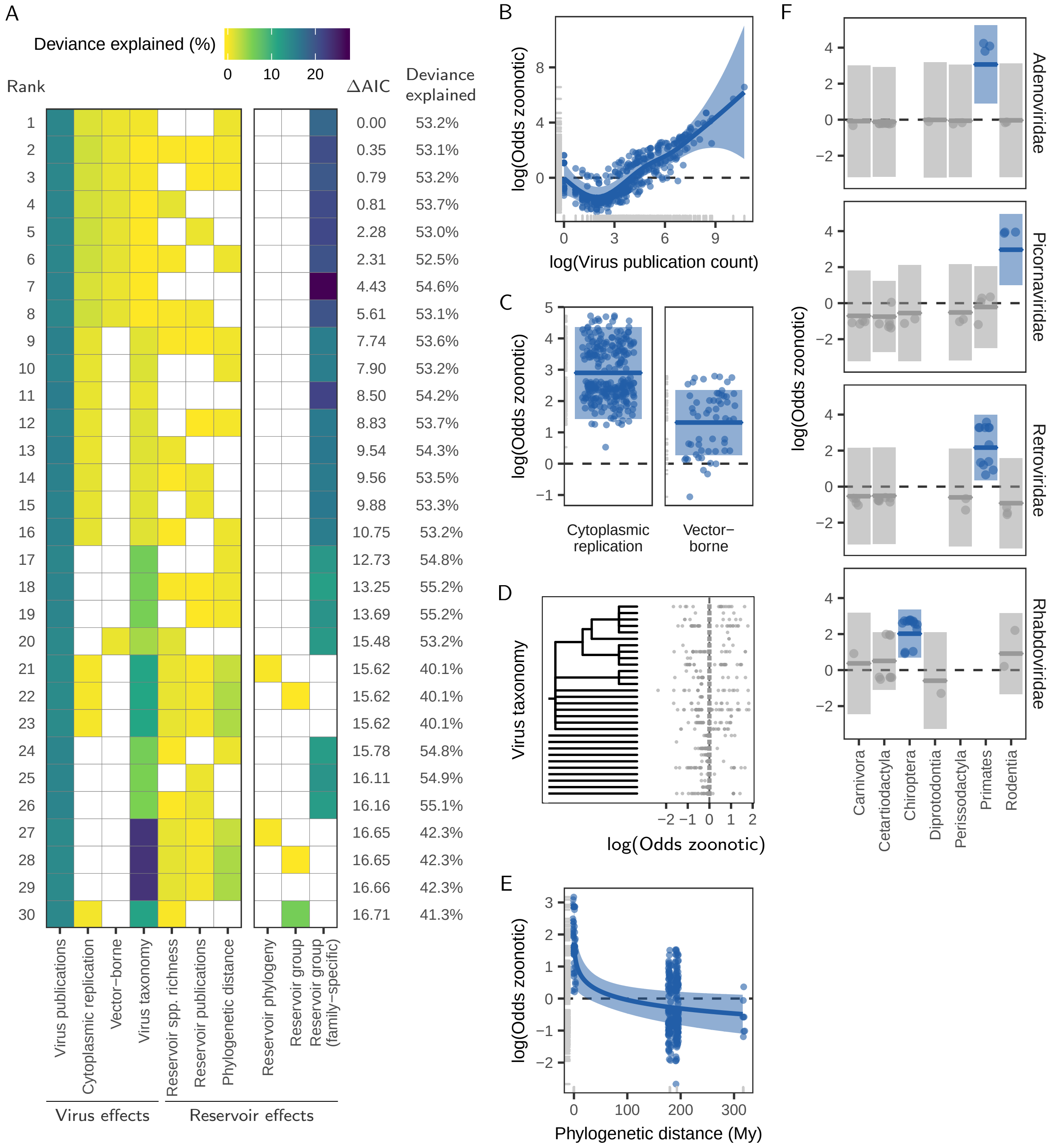

Fig. S7. Predictors of zoonotic propensity when using data from mammalian-associated viruses only. The top 30 models ranked by AIC are shown in (A), with rows representing individual models and columns representing variables. The effects present in the top model are illustrated in panels B – F, with lines indicating the predicted effect of the variable on the x-axis, when keeping all other variables at their mean observed value, and shaded regions indicating the 95% confidence interval of this prediction. Points illustrate partial residuals. Effects whose 95% confidence interval cross 0 over the entire range of the predictor variable are shaded in grey.

References

1. KJ Olival, et al., Host and viral traits predict zoonotic spillover from mammals. *Nature* **546**, 646–650 (2017).
